# Two distinct populations of doublecortin-positive cells in the perilesional zone of cortical infarcts

**DOI:** 10.1186/s12868-015-0160-8

**Published:** 2015-04-15

**Authors:** Albrecht Kunze, Alexandra Achilles, Silke Keiner, Otto W Witte, Christoph Redecker

**Affiliations:** Hans Berger Department of Neurology, Jena University Hospital, Erlanger Allee 101, D-07747 Jena, Germany; Center for Sepsis Control and Care, Jena University Hospital, Jena, Germany

**Keywords:** Doublecortin, Cortex, Neurogenesis, Glia, Ischemia, Photothrombosis

## Abstract

**Background:**

Recovery following stroke depends on cellular plasticity in the perilesional zone (PZ). Doublecortin (DCX), a protein mainly labeling immature neurons in neurogenic niches is also highly expressed in the vicinity of focal cortical infarcts. Notably, the number of DCX+ cells positively correlates with the recovery of functional deficits after stroke though the nature and origin of these cells remains unclear.

**Results:**

In the present study, we aimed to characterize the population of DCX+ cells in the vicinity of ischemic infarcts in a mouse model in detail. Employing a photothrombosis model, distinct immunohistochemical techniques, stereology and confocal microscopy, we show that: i) DCX+ cells in the perilesional zone do not constitute a homogenous population and two cell types, stellate and polar cells can be distinguished according to their morphology. ii) Stellate cells are mainly located in the lateral and medial vicinity of the insult and express astrocytic markers. iii) Polar cells are found almost exclusively in the corpus callosum region including in the preserved deep cortical layers close to the subventricular zone (SVZ). Further, they do not show any colocalisation of glial markers. Polar morphology and distribution suggest a migration towards the lesion.

**Conclusions:**

In summary, our findings provide evidence that in mice DCX+ cells in the perilesional zone of cortical infarcts comprise a distinct cell population and the majority of cells are of glial nature.

**Electronic supplementary material:**

The online version of this article (doi:10.1186/s12868-015-0160-8) contains supplementary material, which is available to authorized users.

## Background

The protein doublecortin (DCX) plays a key role in neuronal migration during development by binding and stabilizing microtubules as cells migrate towards the brain surface. Mutations in DCX induce marked migration defects in humans leading to subcortical band heterotopia (SBH, ‘double cortex’ syndrome) in females and to the more severe lissencephaly (‘smooth brain’ syndrome) in males [1,2]. In the adult brain, DCX is still expressed by immature neurons within the neurogenic niches, in the dentate gyrus (DG) and in the subventricular zone (SVZ). DCX expression in the DG correlates with the extent of adult neurogenesis [3] and DCX+ neurons are required for successful acquisition of spatial learning [4]. Hence, DCX has been widely used as marker of neuronal precursor cells and neurogenesis.

*Following stroke*, DCX was found to be highly expressed in the perilesional cortex of focal ischemic infarcts - beyond the circumscribed neurogenic regions [5,6]. Notably, the numbers of DCX+ cells in the infarct vicinity positively correlate with the recovery of functional deficits [7]. In contrast, transgenic conditional ablation of DCX worsens stroke outcome both in the short and long term on a consistent basis [8,9]. However, it remains unclear which cells in the perilesional zone (PZ) express DCX. A few studies provide evidence of neuroblast migration from the subventricular zone as well as of the rostral migratory stream towards the lesion [5,10,11]. But both the extent and distribution of DCX expression raise doubts on whether the majority of DCX+ cells in the infarct vicinity actually migrate from the SVZ. It is more likely that local residing cells (e.g. neuronal or glial cells) are stimulated to express DCX following ischemia.

The goal of the present study was to characterize the perilesional DCX+ cells in detail with regard to the following three considerations; 1. Do cells show a distinct morphology and distribution? 2. Whether the cells express different immunhistochemical markers, and if so which ones? 3. What is the time course of DCX expression?

Using a focal ischemia model in mice, we demonstrate the presence of two subpopulations of DCX+ cells in the perilesional zone. The more abundant DCX+ cell type reveals a stellate morphology, coexpression of GFAP and S100B and a similar distribution in the perilesional cortex and the corpus callosum – region (CC-region). The other DCX+ cell type with a stronger DCX-expression has fewer processes with polar orientation, is mainly located in CC-region, and does not show any expression of glial markers. Our findings provide evidence that in a murine model of focal ischemia, the majority of DCX+ cells in the perilesional zone are of glial nature.

## Results

### Morphology of the photothrombotic infarcts

All animals analyzed in the experimental subgroups showed typical cortical infarcts in the sensorimotor cortex. The cortical infarct affected all cortical layers leaving the underlying white matter in the majority of animals intact. The mediolateral diameter of the lesions was 1.2 ± 0.4 mm (n = 10).

### Identification of two DCX+ cell populations in the perilesional zone

**Figure 1:**
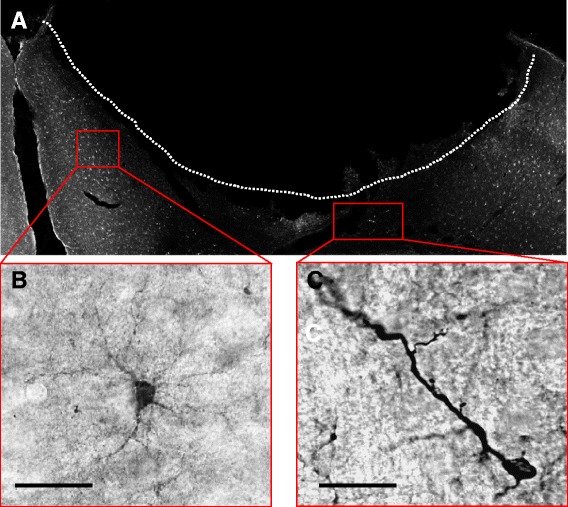
Two distinct DCX+ cells can be distinguished in the PZ. Representative images of 40 μm brain sections of C57 mice following immunohistochemical staining of DCX. **(A)**, Overview of the circumscribed cortical ischemic infarct. **(B)**, Example of DCX+ cell with multiple processes and weaker immunoreactivity, termed DCX+ stellate cells. **(C)** The second DCX+ cell type revealed a polar morphology with one or two processes, note the higher signal intensity of DCX. Scale bars 20 μm.

**Figure 2:**
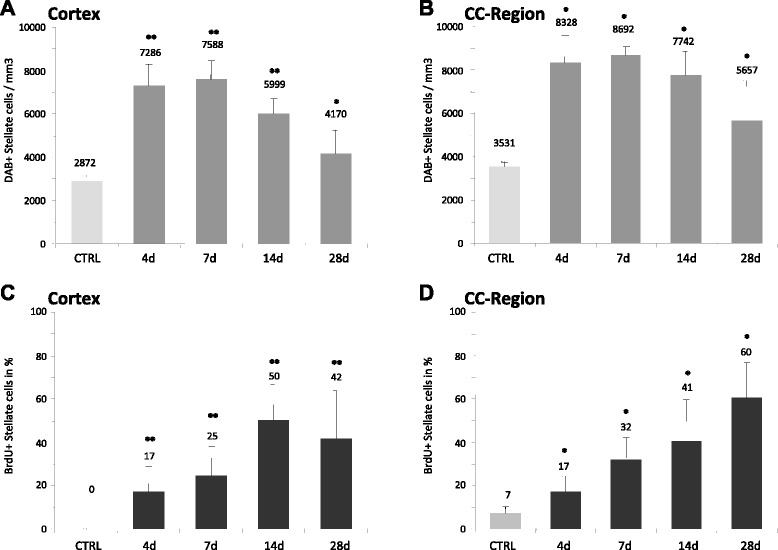
Quantification of DCX+ stellate cells in the PZ. The number of DCX+ cells was quantified using semiautomatic stereology system and the optical-fractionator method. **(A)**, **(B)**, Total numbers of DCX+ stellate cells in the cortex and CC-Region, respectively. **(C)**, **(D)**, Percentage of DCX + stellate cells expressing the proliferation marker BrdU at different time points. Notably, the number of DCX+ stellate cells is similarly increased within both regions following ischemia. Bars represent Mean ± SD. Significant differences were indicated as follows: **(p < 0,01), *(p < 0,05).

Our morphological analysis of randomly chosen DCX+ cells (n = 30) in the perilesional zone according to shape and, in particular according to the number of processes and their orientation showed two distinct DCX+ cell subpopulations (Figure 1). One cell type, which we termed as the DCX+ stellate cell revealed several processes radially extending from the soma. The second cell type had only one or two processes with a strict polar orientation and we named this cell type, the DCX+ polar cell. Intensity of DCX-expression was consistently stronger in the DCX+ polar cells. In the next step, we quantified the two DCX+ cell populations in the PZ. In particular, we aimed to determine whether both cell types revealed differences in abundance and spatial distribution in the infarct vicinity. To this effect we defined different regions of the PZ as follows: i) the medial cortex, ii) the lateral cortex, summarized as the cortex-region, and iii) the corpus callosum - region (or the CC-region comprising the corpus callosum and preserved deep cortical layers). Quantification using a semiautomatic stereological system showed that the number of DCX+ stellate cells were significantly increased by a factor of 2.54 in the *cortex-region* of the PZ at day 4 (7286 ± 1009 versus 2872 ± 246 cells/mm^3^, p < 0.01) (Figure 2). In comparison to the contralateral side, the number of DCX+ stellate cells remained persistently elevated with a slight decrease at day 14 and 28 (Day 7: 2.54-fold, day 14: 2.1-fold, day 28: 1.45-fold). No differences were detected between the medial and lateral cortex of the PZ. Thus, both regions were summarized as the cortex region of the PZ. Notably, the number of DCX+ stellate cells in the contralateral side did not change over time. In the *CC-region*, we observed an increase of DCX+ stellate cells to a similar extent to that in the cortex-region of the PZ. Quantification yielded a 2.36-fold elevation of these cells (8328 ± 1243 vs 3531 ± 234 cells/mm^3^, p < 0.01) at day 4 (Figure 2). The increase was persistent with a decline over time at other time points, (Day 7: 2.46-fold, p < 0.05, Day 14: 2.19-fold, p < 0.05, Day 28: 1.60-fold, p < 0.05).

**Figure 3:**
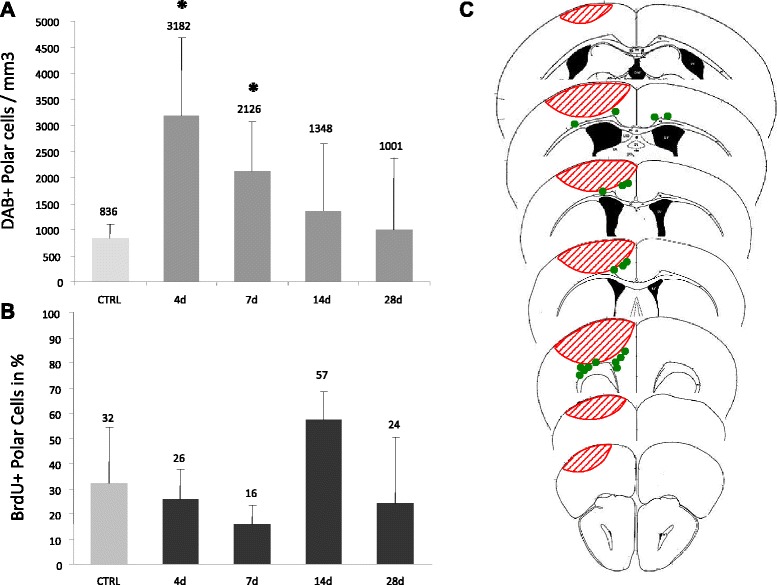
Quantification of DCX+ polar cells in the PZ. **(A)** Total numbers of DCX+ polar cells in CC-Region. **(B)**, Percentage of DCX + polar cells expressing BrdU. **(C)**, Green dots illustrate where polar cells were located in one representative animal. The counting was carried out manually because of the low numbers of polar cells. Bars represent Mean ± SD. Asterisks indicate significant differences: *(p < 0,05).

DCX+ polar cells were detected only in the CC-Region and not observed in the cortex-region even after repeated scanning of the sections by two different investigators (Figure 3). DCX+ polar cells were significantly increased at early time points in the CC-Region compared to the contralateral side (Day 4: 3.8-fold, p < 0.05, Day 7: 2.54-fold, p < 0.05, Day 14: 1.61-fold, n.s.; Day 28: 1.20-fold, n.s.) (Figure 3).

In **summary,** DCX+ stellate cells were seen more frequently in the perilesional zone, revealed a similar distribution in both the cortex- and CC-Regions, and were persistently increased at all time points. In contrast, DCX+ polar cells were detected only within the CC-Region and found increased particularly at early time points in comparison to the contralateral side.

### DCX+ stellate cells express glial markers whilst polar cells do not

Characterisation of the proliferative activity of the two cell populations by BrdU labeling showed that 17% of *DCX+ stellate cells* were BrdU-positive at day 4 in the cortex- and CC-regions, respectively. At the latter time points (Day 7 to 28), BrdU-labeling of the DCX-stellate cells increased, indicating ongoing proliferative activity after day 4 (Figure 2). Analysis of the proliferation marker in the *DCX+ polar cells* revealed a coexpression of BrdU in 26% of the cells at day 4 (Figure 3). However, with respect to BrdU labeling, there was no difference to the contraleral side where 32% of DCX+/BrdU+ cells were also seen. Compared to controls, numbers of BrdU+/*DCX+ polar cells* did not differ significantly at the other time points. Thus, proliferation appeared to be increased especially in the stellate cells of the ipsilateral hemisphere.

**Figure 4:**
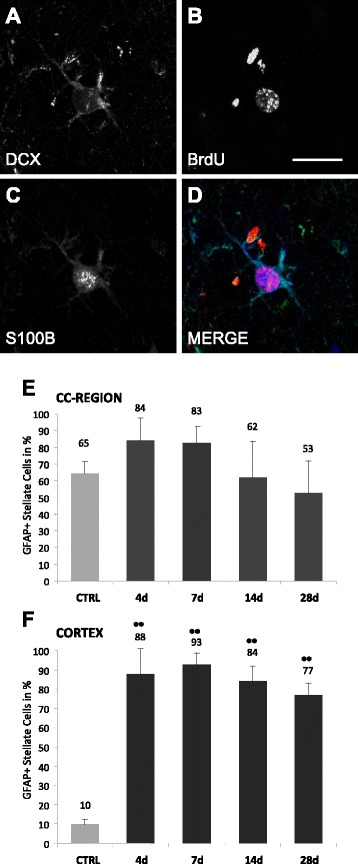
Coexpression of glial markers by DCX+ stellate cells. **(A-D)** Confocal images of single DCX+ stellate cell expressing S100beta. **(E, F)** Quantification of GFAP expression by DCX+ stellate cells in the cortex- and CC-region, respectively. Bars represent Mean ± SD. Significant differences were indicated as follows: **(p < 0,01), *(p < 0,05). Scale bar 20 μm.

Coexpression studies of other cytochemical markers yielded the following results: >80% of DCX-stellate cells coexpressed the glial markers GFAP and S100B whilst overlap of GFAP and S100 B expression was almost complete (Figure 4). In contrast, DCX+ polar cells expressed neither glial proteins nor other markers investigated in the study (Table 1). Notably, both cell types revealed no colocalisation with the mature neuronal marker (NeuN).

**Table 1:** **Summary of cell markers expressed by DCX+ stellate and polar cells**

**Marker**	**Stellate cells**	**Polar cells**
DCX	**+**	**+**
BrdU	**+**	**+**
GFAP	**+**	**-**
S 100 β	**+**	**-**
NeuN	**-**	**-**
Pax6	**-**	**-**
Sox2	**-**	**-**
CNPase	**-**	**-**
Iba 1	**-**	**-**
CD 68	**-**	**-**
DCLK	**-**	**-**

Immunohistochemical Analysis was performed by confocal microscopy studies of double or triple labelled sections. The distinct markers are considered to characterize the following cell types or development stages, BrdU: Thymidine analogon labelling the proliferating cells. GFAP and S100beta: Astrocytes. NeuN: Mature neurons. Pax6 and Sox2: Precursor cells. CNPase: Oligodendrocytes. Iba1 and CD68: Microglia. DCLK: Radial glia and neuronal precursor cells.

We further analyzed the expression pattern of doublecortin-like (DCL) protein by using specific antibodies provided by Bjarte Havik, Bergen, Norway. Herein, we found no DCL expression in either DCX+ stellate or in DCX+ polar cells, respectively. The doublecortin-like (DCL) protein is a splicing variant of the doublecortin-like kinase (DCLK1) which shares 73% amino acid identity with DCX over its entire length of 362 amino acids and also has two DCX domains [12]. DCL is expressed in radial glia-like cells (RGC) during embryogenesis and neuronal precursor cells in the adult SVZ [13].

Finally, there have been recent reports stating that different primary AB might yield variable DCX staining patterns [14]. Herein and in previous experiments, we primarily used the C18 AB (Santa Cruz Biotechnology) that is specific against the carboxyl terminus of DCX protein and revealed reliable intense staining in neurogenic regions [7,15] as well as in the cortex [16]. Our data is in line with the findings of other groups who analyzed the specificity of C18 AB to DCX [14,17]. However, to verify our results we performed a series of test staining with a different DCX antibody (Millipore, Temecula, CA, USA) which did no differ in its staining patterns.

Taken together our findings suggest that the majority of DCX+ stellate cells are of glial origin and that DCX+ polar cells might constitute migrating precursors.

## Discussion

DCX has been identified as a microtubule-associated protein expressed by migrating neuroblasts during a limited phase of their growth in both developing and adult mammals [18-20]. DCX plays a crucial role in neuronal migration by virtue of being involved in microtubule stabilization [19,21], nuclear translocation [22] and growth cone dynamics [22-24].

In the adult normal brain, expression of DCX is rare exterior to the neurogenic areas of the hippocampal formation and subventricular zone. Brown et al. found only one DCX+ cell in every 20–25 sections of normal adult rat neocortex [17]. Under pathophysiological conditions, such as in epilepsy, the expression patterns of DCX are altered and DCX+ cells have been observed in distinct cortical areas [14]. In addition, the DCX+ cell could be detected within the striatum and cortex following ischemia [5,10]. In line with these findings, we showed in a previous study that a major subset of proliferating BrdU+ cells coexpressed DCX in the cortical perilesional zone two weeks after focal infarct [16]. Apparently these cells did not give rise to neurons since observed no BrdU+/NeuN+ cells at a latter time point, i.e. six weeks following ischemia. Thus, the nature of DCX+ cells in the ischemic cortex remains elusive.

Herein, we demonstrate that DCX+ cells in the PZ do not constitute a homogenous cell population but comprise two distinct DCX+ cell types. The more abundant DCX+ cell type revealed a stellate morphology, was homogenously distributed in the PZ (Cortex and CC-Region) and typically expressed glial markers. The other DCX+ cell type was characterized by polar morphology with one or two processes. In addition, we detected no co-labeling with the mature neuronal marker NeuN in both DCX+ cell types on a constant basis at the different time points, indicating that DCX+ cells do not differentiate into neurons in the PZ. Taken together, our results strongly suggest that the majority of DCX+ cells in the perilesional murine cortex are of glial nature.

DCX is generally considered to be a reliable marker of immature neurons and neurogenesis [3,5,25,26]. Our results challenge this assumption, in particular with regard to the lesioned brain exterior to the neurogenic regions. Importantly, other authors have also documented DCX expression in glial cells in previous studies. For example, in an early report, Nacher et al. described DCX+ cells in brain parenchyma that showedthe morphology of astrocytes [27]. In addition, Verwer et al. (2007) reported DCX+ astrocytes in the neocortex of post-mortem human brain tissue. Their data indicate that differentiated astrocytes in the human neocortex associated with various disease backgrounds may contain DCX at levels around the detection threshold [28]. Further, Bloch et al. (2011) analyzed the expression pattern of DCX and colocalization with distinct cell markers in the cerebral cortex of primates and humans. They found a significant number of cortically located cells expressing DCX. 26% of the DCX+ cells coexpressed GFAP. Notably, they confirmed their findings by comparing three different sources of antibodies against DCX [29]. However, the significance of glial DCX expression still remains speculative. Verwer et al. have suggested a function of DCX in the glia-to-neuron communication. It is also conceivable that DCX plays a role in the migration of astrocytes following insults. Alternately, DCX expression might indicate transdifferentiation of mature astrocytes towards a more plastic “stem cell like” phenotype. Our previous findings demonstrating that recovery following stroke correlates with the DCX expression in the PZ [7] suggest a considerable role of DCX in the reorganization of the ischemic brain. However, future studies are necessary to address these questions.

The second DCX+ cell type with a clear polar orientation was almost exclusively detectable in the CC-Region close to the subventricular zone. In contrast to the stellate cells, DCX+ polar cells did not express any glial protein or other markers analyzed in the current study (Table 1). The morphology and location of the DCX+ cells are suggestive of a precursor cell migrating from the subventricular zone. The DCX+ polar cells also appeared to be much brighter in immunofluorescence studies indicating a higher level of DCX expression. In line with previous findings, the stronger DCX signal might be another hint that the DCX+ polar cells are possibly migrating precursor cells [30]. However, we were unable to directly prove the migration of polar cells from SVZ to the lesion because we did not perform lineage tracing experiments.

Finally, some authors report a coexpression of NG2 chondroitin sulfate proteoglycan (NG2) by DCX+ cells in the cortex [31]. NG2 cells comprise the major proliferating cell population in the adult brain. They are mainly considered to be oligodendrocyte progenitors. Although, there is an ongoing debate as to whether, under certain conditions, NG2 cells display features of multipotent precursor cells and give rise to neurons [32,33]. Here, we did not perform co-labeling of DCX and NG2 which might be considered as one limitation of our study. However, in this study our focus was not on oligodendrogenesis but rather on the analysis of the co-expression of other glial markers by DCX-positive cells.

In summary, our study sheds more light on the DCX+ cell population in the perilesional zone of ischemic infarcts in mice. It challenges the view that doublecortin is a specific marker of cells committed to neuronal lineage in particular outside of the neurogenic regions in the lesioned brain. Further studies are required to elucidate the functional role of the distinct DCX+ cells following ischemia.

## Conclusion

Taken together, our study provides evidence that in mice DCX+ cells in the perilesional zone of focal cortical infarcts comprise two distinct cell populations. According to their morphology and marker expression the majority of DCX+ cells have a glial nature.

## Methods

The study was performed on a total number of 32 adult male C57BL/6 J mice (11 to 15 weeks of age, 20–30 g). Animals were held under standard housing conditions in ordinary cages (5animals/cage). Food and water were provided ad libitum. All experimental procedures were approved by the German Animal Care and Use Committee in accordance with the European Directives.

### Induction of photothrombotic infarcts

28 animals received photothrombotic cortical infarcts in the right sensorimotor forelimb cortex, 4 animals were sham operated. Infarcts were induced using the method initially described by Watson [34], adapted for mice by Schroeter [35] and modified partially. Briefly, animals were anesthetized using 1.5 − 2.5% isoflurane in a mixture of oxygen/nitrous oxide (20%/60%). A fiber optic bundle with a 1.8 mm aperture diameter was connected to a cold light source (KL 1500, Schott, Jena, Germany), positioned on the skull 1.0 mm anterior to bregma and 1.8 mm lateral to midline, and adjusted to the stereotaxic coordinates of the forelimb sensorimotor cortex (Paxinos, 2001). The light was turned on for 15 min. Three repetitive injections of 0.1 ml Rose Bengal (10 mg/ml in 0.9% NaCl; Sigma-Aldrich, Taufkirchen, Germany) were given at an interval of 5 min, starting 5 min before light onset. After surgery, the animals were returned to their cages and placed under 12 h light/12 h dark conditions.

### Experimental design

All mice received two intraperitoneal injection of 5-bromo-2-deoxyuridine (BrdU, 50 mg/kg body weight) daily with a two hour gap over four sequential days after infarct induction and were allocated to four experimental groups with different survival times (4, 7, 14 or 28 days, Additional file 1: Figure S1).

### Tissue preparation and immunocytochemistry

The animals were deeply anesthetized with diethylether and perfused through the ascending aorta with 4% paraformaldehyde in phosphate buffer (0.15 mol/L, pH 7.4). Brains were removed immediately after perfusion and postfixed in 4% paraformaldehyde in phosphate buffer overnight. All samples were then cryoprotected in phosphate-buffered saline containing 30% sucrose for 24 h and stored at - 75°C for further processing. Sequential sections were cut using a freezing microtome (40 lm) and stored at - 20°C until further processing. Free-floating 40 μm sections were treated with 0.6% H_2_O_2_ in Tris-buffered saline (0.15 M NaCl, 0.1 M TrisH_2_O_2_ in Tris-buffered saline (0.15 M NaCl, 0.1 M Tris–HCl, pH 7.5) for 30 min to block endogenous peroxidase. After washing, the sections were incubated in 2 N HCl at 37°C for 30 min. Sections were rinsed in boric acid (pH 8.5) and after several rinses in Tris-buffered saline containing 0.25% Triton X-100 (Tris-Triton), sections were incubated overnight at 4°C in primary goat polyclonal or guinea pig polyclonal anti-Doublecortin (1:200) in TBS-Triton containing 5% normal donkey serum or 5% goat serum. The next day, sections were incubated in biotinylated donkey anti goat or anti guinea pig antisera (1:500, Jackson Immunoresearch, West Grove, PA) for 2 h, rinsed and incubated in avidin-biotin-peroxidase complex (Vector Laboratories, Burlingame, CA) for 60 min. Finally, they were reacted for peroxidase detection in a solution of 3.3-diaminobenzidine (0.25 mg/mL; Sigma- Aldrich, Munich, Germany) containing 0.01% H_2_O_2_. Sections were thoroughly washed, mounted, and coverslipped. For double or triple immunofluorescence staining, the brains were treated in the same way as described for immunoperoxidase staining aside from pretreatment with Tris-H_2_O_2_. Sections were then incubated with different primary antibodies (Additional file 2: Table S1) for 24 hours, followed by incubation for 48 hours with secondary antibodies labelled with distinct fluorochromes (Additional file 2: Table S1). Sections were rinsed again, mounted onto gelatin-coated slides and coverslipped in polyvinyl alcohol with diazabicyclo-octane (DABCO) as antifading agent. For double or triple immunofluorescence stainings, we applied the following combinations of primary antibodies: DCX-BrdU-NeuN, DCX-BrdU-GFAP, DCX-BrdU-S100B, DCX-Sox2, DCX-Pax6, DCX-CNPase, DCX-CD68-IbA, DCX-DCLK.

### Quantification and statistical analysis

To quantify DCX-labeled cells with a stellate morphology in the perilesional area, the optical fractionator method as a semi-automatic stereological system was used. The perilesional zone was defined as an approximately 350 μm wide frame that directly surrounding the infarct (Additional file 1: Figure S1). The following stereological parameters were used: 80 × 80 μm^2^ counting frame and a 100 × 180 μm^2^ sampling grid. Section thicknesses were measured and appropriate guard zones at the top and the bottom of the section were defined to avoid over sampling. The boundaries of the perilesional areas were delineated using a 5× objective and the numbers of the DCX positive cells (peroxidase method) were counted at a magnification of 63 ×. Positive cells, which intersected the uppermost focal plane and the lateral exclusion boundaries of the counting frame were not counted. BrdU counts were calculated as absolute numbers per mm^3^ sectional volume. To quantify the DCX-labelled polar cells, the perilesional zone was defined in the same way but the counting was carried out manually because of the low numbers of polar cells. To phenotype the DCX-positive cells, coexpression of other cell specific markers (Table 1) were analyzed by confocal laser scanning microscopy (LSM Meta 710; Zeiss, Germany). For each animal, about 50 DCX-positive stellate cells and all detectable polar cells were colocalized employing z-stacks. The absolute numbers of colabeled cells were calculated per animal by multiplying the absolute number of DCX-positive stellate cells with the percentage of cell marker of interest. In general, every 12^th^ brain section was used for quantification or phenotyping of DCX-positive cells by confocal laser microscopy. The intensity of DCX expression was assessed by visual control by two independent investigators who categorized the cells as being weak or bright fluorescent.

Statistical analyses were performed using SPSS 19.0 for Windows. Differences were assessed with the Mann–Whitney U test. Data are given as mean ± SD unless otherwise noted. P values < 0.05 were considered statistically significant.

## Additional files

Additional file 1: Figure S1. Illustration of experimental design. Additional file 2: Table S1. Primary antibodies and secondary antibodies used for immunohistochemistry.
