# Supplementary figures and images for: Two distinct populations of doublecortin-positive cells in the perilesional zone of cortical infarcts
Source: BMC Neurosci. 2015 Apr 15;16:20. doi: 10.1186/s12868-015-0160-8 (PMC4404690; doi:10.1186/s12868-015-0160-8)

**A**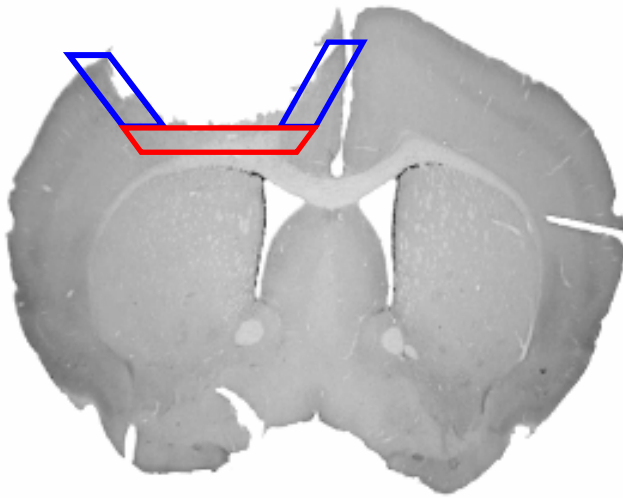**B**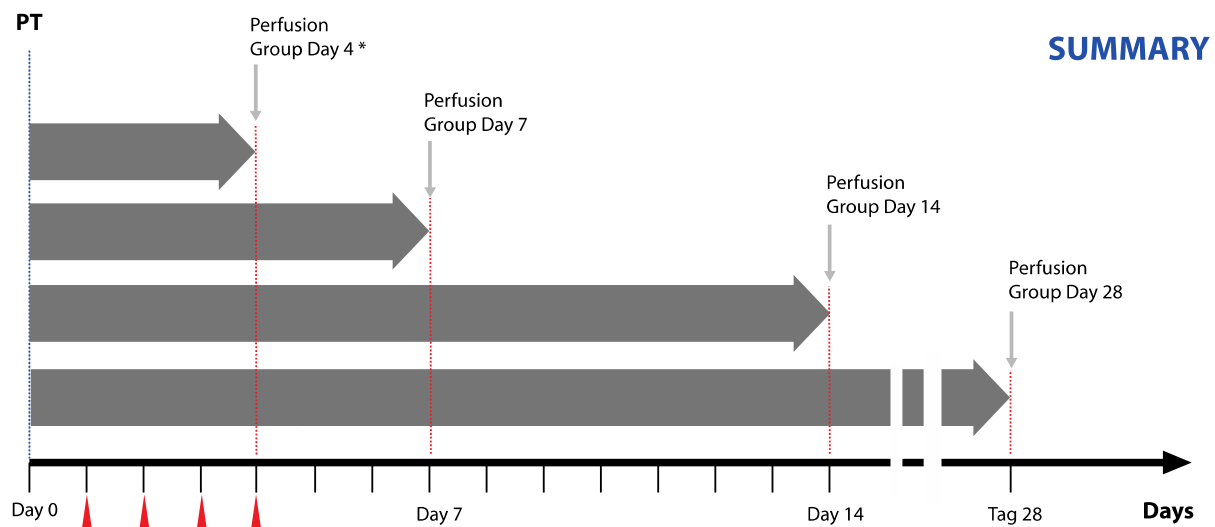

BrdU  
(ip injection 2x per day from  
day 1 to 4, dose 50mg/kg)

\* 2h following the last BrdU injection

**PT**

**Day 4**

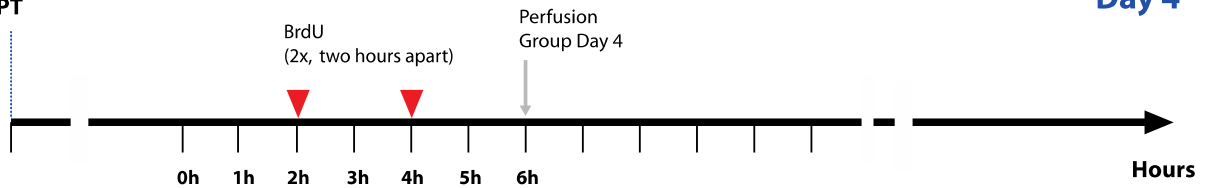

Supplement: Additional file 1: Figure S1. — Illustration of experimental design. [file 12868_2015_160_MOESM1_ESM.pdf]
