# Supplementary material for: Two distinct populations of doublecortin-positive cells in the perilesional zone of cortical infarcts
Source: BMC Neurosci. 2015 Apr 15;16:20. doi: 10.1186/s12868-015-0160-8 (PMC4404690; doi:10.1186/s12868-015-0160-8)
Supplement: Additional file 2: Table S1. — Primary antibodies and secondary antibodies used for immunohistochemistry. [file 12868_2015_160_MOESM2_ESM.pdf]

Supplemental Information

Table 1: Primary antibodies and secondary antibodies used for immunohistochemistry

**Primary antibody**	**Made in**	**Dilution**	**Source**	**Cell type labeled**
Anti-BrdU	rat	1:500	AbD Serotec Düsseldorf, Germany	Cells in S-phase when BrdU is injected
Anti-GFAP	mouse	1:500	Millipore, Temecula, CA, USA	Astrocytes
Anti-S100β	rabbit	1:2000	Swant, Bellizona, Switzerland	Astrocytes
Anti-CD68	mouse	1:500	Serotec, Oxford, UK	Microglia
Anti-DCX	goat	1:500	Santa Cruz Biotechnology, Santa Cruz, CA, USA	Immature neurons, Neuronal progenitors
Anti-DCX	guinea-pig	1:200	Millipore, Temecula, CA, USA	Immature neurons, Neuronal progenitors
Anti-NG2	rabbit	1 :200	Millipore, Temecula, CA, USA	Glial cells, Oligodendrocyte progenitors
Anti-Sox2	rabbit	1 :1500	Abcam, Cambridge Science Park, UK	Adult neural stem cells, neuronal progenitors
Anti-CNPase	mouse	1:500	Abcam, Cambridge Science Park, UK	Oligodendrocytes
Anti-Pax6	rabbit	1.500	Covance, Berkeley, CA, USA	Neuronal progenitor cells
Anti-DCL	rabbit	1:1000	Provided by Bjarte Harvik, Bergen, Norway	Neuronal progenitor cells
Anti-NeuN	mouse	1:500	Millipore, Temecula, CA, USA	Mature neurons

**Secondary antibody**	**Made in**	**Dilution**	**Source**	**Fluorescent conjugate**
Anti-rat	donkey	1:500	Dianova, Hamburg, Germany	Rhodamin Red-X
Anti-mouse	donkey	1:500	Molecular Probes, Leiden, Netherlands	Alexa Fluor 488
Anti-goat	donkey	1:250	Molecular Probes, Leiden, Netherlands	Alexa Fluor 488
Anti-guinea-pig	goat	1:500	Molecular Probes, Leiden, Netherlands	Alexa Fluor 488

Anti-goat	donkey	1:500	Dianova, Hamburg, Germany	CY5
Anti-rabbit	donkey	1:500	Dianova, Hamburg, Germany	CY5
